# *Bacillus subtilis* genome vector-based complete manipulation and reconstruction of genomic DNA for mouse transgenesis

**DOI:** 10.1186/1471-2164-14-300

**Published:** 2013-05-03

**Authors:** Tetsuo Iwata, Shinya Kaneko, Yuh Shiwa, Takayuki Enomoto, Hirofumi Yoshikawa, Junji Hirota

**Affiliations:** 1 Department of Bioengineering, Graduate School of Bioscience and Bioengineering, Tokyo Institute of Technology, Yokohama, 226-8501, Japan; 2 Department of Molecular Bioscience, Graduate School of Bioscience and Bioengineering, Tokyo Institute of Technology, Yokohama, 226-8501, Japan; 3 Genome Research Center, NODAI Research Institute, Tokyo University of Agriculture, Tokyo, 156-8502, Japan; 4 Center for Biological Resources and Informatics, Tokyo Institute of Technology, 4259-B63 Nagatsuta-cho, Midori-ku, Yokohama, 226-8501, Japan; 5 Department of Bioscience, Tokyo University of Agriculture, Tokyo, 156-8502, Japan

## Abstract

**Background:**

The *Bacillus subtilis* genome (BGM) vector is a novel cloning system for large DNA fragments, in which the entire 4.2 Mb genome of *B. subtilis* functions as a vector. The BGM vector system has several attractive properties, such as a large cloning capacity of over 3 Mb, stable propagation of cloned DNA and various modification strategies using RecA-mediated homologous recombination. However, genetic modifications using the BGM vector system have not been fully established, and this system has not been applied to transgenesis. In this study, we developed important additions to the genetic modification methods of the BGM vector system. To explore the potential of the BGM vector, we focused on the fish-like odorant receptor (class I OR) gene family, which consists of 158 genes and forms a single gene cluster. Although a *cis*-acting locus control region is expected to regulate transcription, this has not yet been determined experimentally.

**Results:**

Using two contiguous bacterial artificial chromosome clones containing several class I OR genes, we constructed two transgenes in the BGM vector by inserting a reporter gene cassette into one class I OR gene. Because they were oriented in opposite directions, we performed an inversion modification to align their orientation and then fused them to enlarge the genomic structure. DNA sequencing revealed that no mutations occurred during gene manipulations with the BGM vector. We further demonstrated that the modified, reconstructed genomic DNA fragments could be used to generate transgenic mice. Transgenic mice carrying the enlarged transgene recapitulated the expression and axonal projection patterns of the target class I OR gene in the main olfactory system.

**Conclusion:**

We offer a complete genetic modification method for the BGM vector system, including insertion, deletion, inversion and fusion, to engineer genomic DNA fragments without any trace of modifications. In addition, we demonstrate that this system can be used for mouse transgenesis. Thus, the BGM vector system can be an alternative platform for engineering large DNA fragments in addition to conventional systems such as bacterial and yeast artificial chromosomes. Using this system, we provide the first experimental evidence of a *cis*-acting element for a class I OR gene.

## Background

Technological developments in chromosome engineering are essential for the manipulation and functional analysis of genomic DNA fragments. Artificial chromosomes, such as bacterial artificial chromosomes (BACs) [1] and yeast artificial chromosomes (YACs) [2], have been used for these purposes in combination with transgenesis. BAC and YAC transgenesis techniques have contributed greatly to genome research. However, there are several technological limitations in their cloning size, genetic modification and insert stability. BAC clones are easy to manipulate and retrieve due to their plasmid form and the stability of the cloned DNA. The methods for modifying BAC inserts require additional recombination components, e.g., RecA or phage-derived recombination proteins [3-6]. The BAC system can generally accommodate up to 300 kb genomic inserts. In contrast to the BAC system, genomic DNA inserts of up to 2 Mb can be used with the YAC system. Although the inserts can be easily modified by homologous recombination, YACs often suffer from insert chimerism and unwanted rearrangements due to potent and constitutive yeast recombination activity [7,8]. Generally, the isolation of intact YACs is difficult because of their linear form and contamination with endogenous yeast chromosomes. Thus, these two systems have complementary advantages over each other in terms of cloning capacity and insert stability.

The *Bacillus subtilis* genome (BGM) vector system has been developed as a novel cloning system using a unique concept in which the entire 4.2 Mb genome of *B. subtilis* functions as a vector [9-12]. The cloning strategy for this vector system is based on unique *B. subtilis* features [13,14]. *B. subtilis* expresses competence-related genes at the late stage of cell growth, and their products, transformation machinery molecules, are assembled in the cell membrane. The transformation machinery non-specifically binds and imports extracellular DNA fragments into the cytoplasm in single-stranded form. The recombinogenic DNA is incorporated into the *B. subtilis* genome by homologous recombination. Therefore, if the *B. subtilis* genome is engineered to have homologous cloning site sequences, a target DNA fragment flanked by homologous sequences is easily cloned into the BGM. Special handling of the BGM vector system is not required [12]. *B. subtilis* can be cultivated under the same conditions as *Escherichia coli* in LB broth at 37°C. The competency of *B. subtilis* enables easy transformation procedures and efficient recombination reactions. For instance, competent *B. subtilis* cells can be prepared by merely cultivating the cells in a special medium for several hours, and these cells are transformed by simply mixing in DNA fragments without additional heat-shock or electroporation [12].

The specific features of the BGM vector system include a cloning capacity over 3 Mb [15], the stable propagation of cloned DNA fragments in a single copy per cell and the amenability of various modification strategies based on RecA-mediated homologous recombination [10,16,17]. These advantages make the BGM vector system an attractive alternative for the manipulation of large DNA. It has been used to clone genomic DNA from several species, including cyanobacteria [15], *Arabidopsis*[10] and mouse [17,18]. Modifications consisting of deleting and fusing cloned inserts have also been achieved [16,17]. However, genetic modification methods have not been fully established in the BGM vector system; targeted insertion and inversion modifications remain to be demonstrated, and the fusion of two contiguous DNA fragments is limited to clones that are orientated in the same direction. In addition, the BGM system has not been applied to transgenesis. Thus, the BGM vector system is a developing technology with attractive potential, which includes its megabase-cloning capacity and homologous recombination-based genetic modification.

**Figure 1:**
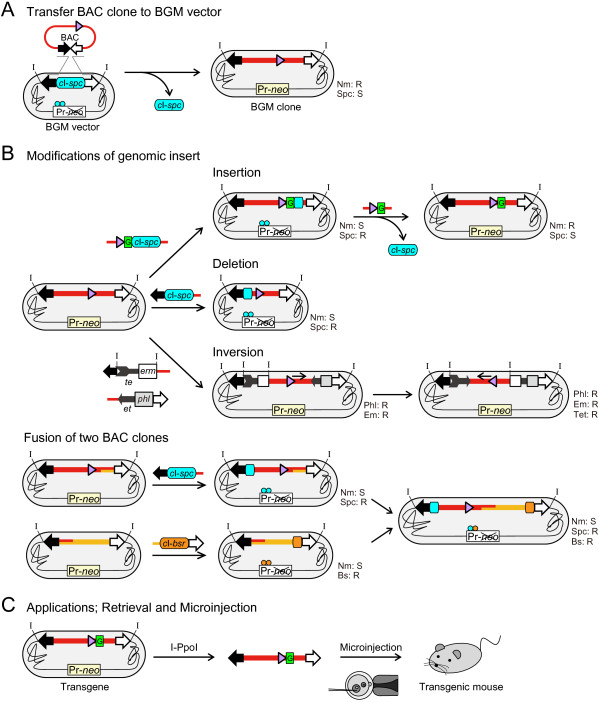
**Cloning and various modification methods of the BGM vector system in the generation of transgenic mice.** (**A**) The cloning strategy used to introduce BAC inserts into the BGM vector (Figure 2). BGM vectors possess two antibiotic gene cassettes: Pr-*neo*, a lambda Pr promoter fused to the neomycin resistance gene (*neo*), and *c*I-*spc*, which contains *c*I encoding the CI repressor protein, which binds to the Pr promoter, fused to the spectinomycin resistance gene (*spc*). The *c*I-*spc* is flanked by two BAC vector sequences that function as the cloning site for BAC inserts. The original BGM strains are resistant to Spc and sensitive to Nm. Once a BAC insert is cloned into the BGM vector, the recombinant becomes resistant to Nm and sensitive to Spc because the *c*I-*spc* cassette is replaced by the BAC insert. (**B**) Modifications of large DNA fragments. The BGM vector system allows various manipulations, including insertion (e.g., EGFP is designated as “G” in the box) (Figure 3), deletion and the inversion (Figure 4) and fusion of two overlapping clones (Figure 5). (**C**) The BGM inserts can be retrieved by several methods, and the recombinant DNA fragments can be used for the generation of transgenic animals by microinjection (Figure 6). The closed triangles represent a target gene and its direction. The BAC vector sequences are indicated by the closed and open arrows. The half part of BAC vector containing chloramphenicol resistance gene for *E. coli* is depicted by open arrow (defined as right side). The I-PpoI sites, designated as “I” are introduced to flank the BAC vector sequences. The deletion method is described in [17].

In this study, we have added two new genetic modifications to the BGM system tool kit. One is targeted insertion of a reporter gene without introducing an unwanted selectable marker at the target site when constructing the transgene. Another is inversion of the cloned insert to align its orientation so that two contiguous BAC clones in opposite directions can be used to reconstruct the genomic structure. In fact, inversion of a cloned insert has not been reported in any other system. To explore the potential utility of the BGM vector in mouse transgenesis, we focused on the fish-like odorant receptor (class I OR) gene family. This OR family is a phylogenetically ancient mammalian OR family [19]. The mouse class I OR gene family consists of 158 genes, forming an approximately 3 Mb gene cluster [20,21]. Although a *cis*-acting locus control region is expected to regulate transcription, such a region has not been found. In this study, we not only demonstrated the ability to use the BGM vector system to manipulate and reconstruct mouse genomic DNA fragments and perform mouse transgenesis (Figure 1), but we also found evidence of a *cis*-acting regulatory element for class I OR gene expression.

## Results

### Cloning of genomic DNA fragments into the BGM vector

**Figure 2:**
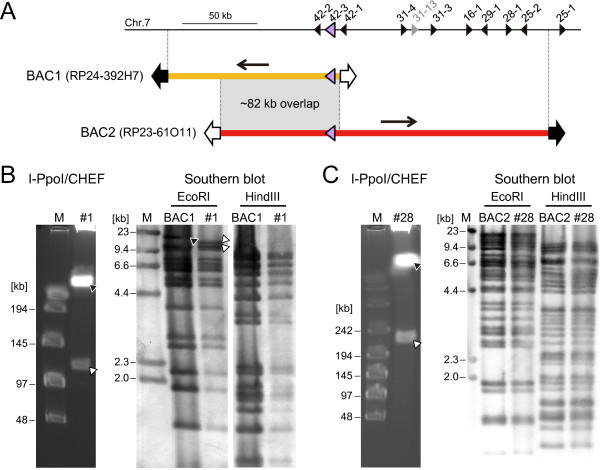
**Cloning of BAC inserts into the BGM vector.** (**A**) Genomic structures of mouse chromosome 7 and the BAC inserts used in this study. The BAC clones cover a portion of the class I OR locus on chromosome 7, share 82 kb overlapping region and are oriented in opposite directions in the BAC vector. A large triangle depicts *MOR42-3*, black triangles indicate other OR genes, and the gray triangle represents the pseudo-OR gene [20]. (**B**, **C**) The cloning of BAC1 and BAC2 into the BGM vector was confirmed by I-PpoI/CHEF (left panels in **B** and **C**) and Southern blotting (right panels in **B** and **C**). The representative BGM vectors with transferred BAC1 and BAC2 inserts were digested with I-PpoI and resolved by CHEF. The open arrowheads indicate BAC insert bands, and the closed arrowheads indicate the BGM vector. Purified original BAC clones and genomic DNA from representative BGM clones were digested with EcoRI or HindIII and hybridized with the original BAC clones as probes. The BGM recombinants showed a band pattern identical to the original BAC clones (lane: BAC), except for the bands corresponding to the BAC end sequences from EcoRI-digested BAC1; the closed arrowhead indicates a BAC-specific signal, and the open arrowheads indicate BGM-specific signals from EcoRI-digested BAC1. In other digestion patterns, the bands of the BAC end sequences were overlapping and indistinguishable from insert signals predicted from restriction maps of BAC clones and the BGM vector. Numbers above lanes are the BGM clone numbers. In lane M, lambda/HindIII fragments or a lambda DNA concatemer was used as a size marker.

Mouse genomic BAC libraries have been constructed that cover nearly the entire mouse genome, and each clone contains a genomic DNA fragment with an average size of approximately 150 kb [22]. The BGM vector system can utilize these valuable DNA resources in a one-step transfer [10]. We prepared two BAC clones, designated BAC1 and BAC2, which overlapped each other via 82 kb region and carried 114 kb and 220 kb mouse genomic DNA fragments containing two and ten class I OR genes, respectively (Figure 2A). We transferred these BAC inserts into BGM vectors, which harbor BAC vector sequences, to clone the BAC inserts by homologous recombination (Figure 1A) [10,16]. Briefly, the original BGM vector is resistant to spectinomycin (Spc) and sensitive to neomycin (Nm) due to repression of the Pr-*neo* cassette by the CI repressor. The BAC inserts taken up by the *B. subtilis* are integrated directly into the genome (BGM vector) via double crossings-over recombination with the BAC vector sequences of the BGM vector. Once a BAC insert is cloned into the BGM vector, the recombinants become resistant to Nm and sensitive to Spc, because the BAC insert replaces the *c*I-*spc* cassette. This selection mechanism can be used in *c*I cassette-mediating modifications, such as the targeted insertion technique. Because two 23-bp sequences “ATGACTCTCTTAA/GGTAGCCAAA” recognized by the rare-cutting endonuclease, I-PpoI are introduced at the both sides of the BAC cloning site, I-PpoI digestion enables to excise out the cloned insert from the BGM vector. The inserts were evaluated by digesting the genomic DNA of the recombinant vectors with I-PpoI followed by contour-clamped homologous electric field (CHEF) gel electrophoresis (Figure 2B, C). Three of seven candidate clones (resistant to Nm and sensitive to Spc) in BAC1 and one of 29 candidate clones in BAC2 contained BAC inserts. The efficiency of cloning the BAC insert into the BGM vector differs between BAC clones used (Additional file 1). We cloned 10 different BAC clones with 3 to 100% efficiency (average 40%). The cloned inserts were further confirmed by Southern blot analyses using the original BAC clones as probes; the results indicated that the digest patterns of the BGM recombinants were identical to those of the original BAC clones, except for the fragments derived from the ends of the insert (Figure 2B, C). Thus, the BGM vector system imported the BAC insert by simple transformation, which enabled the use of already established valuable BAC resources.

### Targeted insertion of a reporter gene

**Figure 3:**
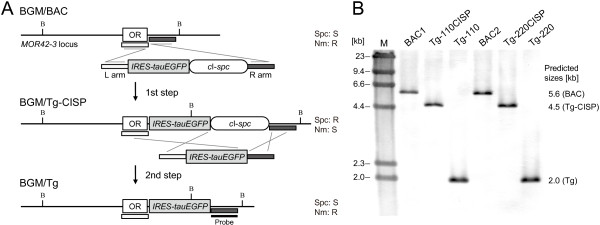
**Targeted insertion of a reporter gene.** (**A**) In the first step, *IRES-tauEGFP* was inserted into the targeted site 3 bp downstream of the *MOR42-3* stop codon by transformation with a purified DNA fragment consisting of L and R arms (1.0 kb each) homologous to *MOR42-3* and the *c*I-*spc* selection cassette. In the second step, the selection cassette was removed by transformation with a fragment lacking the selection marker. (**B**) The targeted insertion of the reporter gene was confirmed by Southern blot analysis using an R homology region as a probe. The genomic DNA of each BGM clone was digested with BamHI and resolved by CHEF, and the changes in the sizes of the BamHI fragments were as expected, indicating the successful targeted insertion of *IRES-tauEGFP*. The sensitivity to antibiotics is shown. R, resistant; S, sensitive. The BamHI sites are represented by “B”. Lane M, lambda/HindIII fragments were used as a size marker.

To construct transgenes for the analysis of *cis*-acting elements of class I OR genes, we inserted an *IRES-tauEGFP* reporter gene cassette [23], which consisted of an internal ribosome entry site and the tauEGFP fusion protein coding sequence, into the class I OR gene *MOR42-3* locus by two-step recombination using the *c*I-*spc* cassette (Figure 3A). In the first step, the BGM clones containing the BAC inserts were transformed with a reporter cassette with the selection marker *c*I-*spc* flanked by 1.0 kb homology regions to the target OR locus (L and R arms) to generate Tg-110CISP and Tg-220CISP. Four of 15 Spc-resistant Tg-110CISP clones and six of 15 Spc-resistant Tg-220CISP clones were sensitive to Nm. In the second step, the counterselection was performed to remove selection marker using a reporter gene cassette without a *c*I-*spc* cassette to generate two transgenes, Tg-110 and Tg-220. Nm-resistant and Spc-sensitive clones were screened by PCR to amplify the region between *EGFP* and R arm. The *c*I-*spc* cassettes were correctly removed in four of 15 tested clones for Tg-110 and two of 48 clones for Tg-220. Negative clones were no PCR product, suggesting that insert DNA sequences had been also deleted along with the *c*I-*spc* cassettes. Relatively low success of the recombination events in the counterselection was previously reported in the BGM system [18] as well as the BAC system [6]. Accordingly, two transgenes, Tg-110 and Tg-220, which were derived from BAC1 and BAC2, respectively, were constructed by inserting *IRES-tauEGFP* 3 bp downstream of the *MOR42-3* stop codon. Southern blot analysis of representative clones with the R arm probe indicated the correct insertion of the reporter cassette (Figure 3B). Thus, we established a targeted insertion method using the BGM vector system with homologous recombination using several antibiotic resistance genes and the *c*I gene without leaving any trace of selection markers or site-specific recombination sites, such as loxP and FRT, in the target sequence (Figure 1B) [5,6].

### Inversion of the insert in the BGM vector

**Figure 4:**
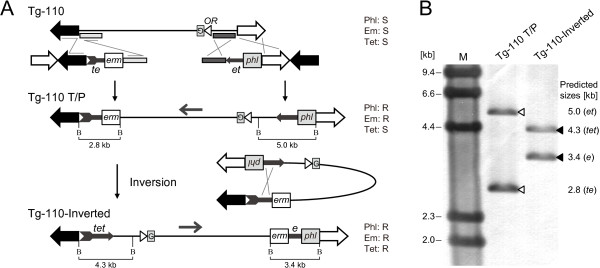
**Inversion of the Tg-110 insert.** (**A**) Schematic diagram of inversion strategy. Two incomplete fragments of the tetracycline resistance gene (*tet*), *te* (5′ side) and *et* (3′ side), which contained an overlapping region *e*, were used for inversion. The inversion cassettes, *phl*-*et*, consisting of *et* and the phleomycin resistance gene (*phl*), and *te-erm*, consisting of *te* and the erythromycin resistance gene (*erm*), were sequentially inserted into the ends of the Tg-110 insert. A 0.9 kb fragment in the left end and a 0.8 kb fragment in the right end were used as homology arms. Homologous recombination between *te* and *et* in Tg-110 T/P results in the inversion of the insert to form an intact *tet* gene. The about positions of BamHI sites are represented by “B”. (**B**) Inversion was confirmed by Southern blotting using the *tet* gene as a probe. The genomic DNA of the represented clones was digested with BamHI. Changes in the size of *tet* cassette fragments represented inversion by intrachromosomal homologous recombination. The open (2.8 kb *te-erm* cassette and 2.3 kb *phl*-*et* plus 2.7 kb Tg-110 fragment) and closed (3.4 kb *erm-e-phl* cassette and 1.6 kb *tet* plus 2.7 kb Tg-110 fragment) arrowheads indicate signals before and after inversion, respectively.

Tg-110 and Tg-220 shared approximately 82 kb of overlapping sequence (Figure 2A), which would enable the fusion of these two fragments to create a longer transgenic construct in the BGM vector system if the two clones were cloned in the same direction. This technique extends one insert by homologous recombination between the overlapping region of another insert and the common vector region [16]. However, the Tg-110 and Tg-220 inserts were cloned in opposite directions in the BGM vector. In this case, the two clones could not be used to extend the insert by the double crossing-over recombination. Insert inversion techniques have not been reported in the BGM or other conventional systems. To fuse these two fragments, we first inverted the Tg-110 insert to orient it in the same direction as Tg-220 using two incomplete fragments of the tetracycline resistance gene (*tet*), *te* (5′ side) and *et* (3′ side), which shared an approximately 1.1 kb overlap region designated *e*[24]. The erythromycin resistance (*erm*) and phleomycin resistance (*phl*) genes were added as selection markers to the *te* and *et* fragments, respectively. The two inversion cassettes, *te-erm* and *phl-et*, were sequentially inserted at the ends of the Tg-110 insert in the reverse direction (Figure 4A). Briefly, *phl-et* cassette was inserted to the right end of the Tg-110 insert by transforming Tg-110 with the linearized BAC plasmid containing the *phl-et* cassette and a 0.8 kb homology region. The *phl-et* cassette was inserted in all five Phl-resistant clones assessed by PCR. Subsequently, we inserted the *te-erm* to the left end of the insert using the linearized BAC plasmid containing the *te-erm* cassette with a 0.9 kb homology region, and obtained Tg-110 T/P (1 clone/1 tested). Intrachromosomal homologous recombination between *te* and *et* via the overlapping region *e* (Tg-110 T/P) resulted in an inversion of the insert and the formation of a complete *tet* gene, enabling the selection of the inverted clone (Tg-110-Inverted) using Tet (Figure 4A). We obtained over 100 Tet-resistant colonies from 20 μl of Tg-110 T/P overnight culture. The formation of *tet* and *erm-e-phl* cassettes was confirmed by PCR in all four randomly picked clones from these colonies. As expected, Southern blot analysis using a *tet* probe revealed changes in size of the *tet* cassette fragments, indicating the successful inversion of the Tg-110 insert (Figure 4B). Because the direction of the inserts is not coordinated in the BAC library, the inversion technique for insert fragments is essential to fuse and reconstruct genomic structures using BAC clones.

### Fusing two inserts to reconstruct genomic structures

**Figure 5:**
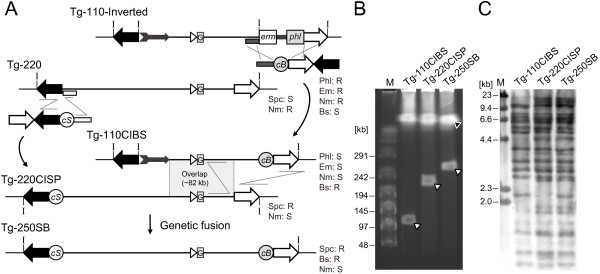
**The genetic fusion of the inverted Tg-110 and Tg-220 fragments to reconstruct the genomic structure of the *MOR42-3 *locus.** (**A**) Schematic diagram of fusion of Tg-110-Inverted and Tg-220 fragments. The selection marker cassettes *c*I-*bsr*, consisting of the *c*I and blasticidin S resistance genes (depicted as *c*B), and *c*I-*spc* (depicted as *c*S) were inserted to the right end of the inverted Tg-110 and to the left end of Tg-220, respectively. 1.3 kb fragments in the left end of Tg-220 and in the right end of Tg-110-Inverted were used as homology arms. Genomic DNA was isolated from the *bsr*-labeled Tg-110 clone (Tg-110CIBS) and added to the *spc*-labeled Tg-220 competent cells (Tg-220CISP). The Tg-220 insert was extended to 252 kb by homologous recombination at the overlap region. (**B**) The fused Tg-250SB insert was evaluated by I-PpoI digestion followed by CHEF. Tg-250SB shows a larger band that corresponds to the fused insert fragment. The closed and open arrowheads represent bands from the 4.2 Mb BGM vector and inserts, respectively. (**C**) The structure of the Tg-250SB insert was confirmed by Southern blot analysis. Genomic DNA was digested with EcoRI and hybridized with Tg-110 and Tg-220 probes. The genetic fusion of the two inserts is shown as the sum of the respective Tg-250SB bands. The I-PpoI sites are represented by “I”. Lane M, lambda/HindIII fragments or a concatemer of lambda DNA was used as a size marker.

We then fused Tg-220 and the inverted Tg-110 to enlarge the mouse genomic DNA fragment (Figure 5A). The *c*I-*spc* and *c*I-*bsr* marker gene cassettes were inserted into the left end of the Tg-220 insert and the right end of the inverted Tg-110 insert, respectively. We transformed Tg-220 with the linearized BAC plasmid containing the *c*I-*spc* and a 1.3 kb homology region, and obtained Tg-220CISP clones (9 clones from 10 Spc-resistant clones). Tg-110-Inverted was similarly transformed with the *c*I-*bsr* cassette fragment containing a 1.3 kb homology region, and obtained Tg-110CIBS clones (2 clones from 2 Bs-resistant clones). The two transgenes were fused by genetic transformation of Tg-220CISP with the purified genomic DNA of Tg-110CIBS. Homologous recombination of the 82 kb overlapping region and the common sequence of the BGM vectors resulted in the extension of the insert to generate Tg-250SB. Twenty-six of 29 Spc- and Bs-resistant colony recombinants were sensitive to Nm. We selected two representative clones for further examinations. The two Tg-250SB clones turned to be sensitive to Tet, whose resistance gene exists in the left end of the Tg-110-Inverted insert and was removed by the fusion, suggesting correct recombination occurs to extend the insert. I-PpoI/CHEF analyses of the Tg-250SB genomic DNA demonstrated a larger band corresponding to the 252 kb fused insert fragment (Figure 5B). Southern blot analysis showed that Tg-250SB was composed of two transgenes, Tg-220 and Tg-110 (Figure 5C), indicating that the two inserts were successfully fused to extend the insert size.

### DNA sequencing of modified and reconstructed BGM clones

To verify the reliability of the BGM vector system in manipulating genomic DNA fragments and its accuracy in genetic engineering, we performed DNA sequencing of the original BAC clones (BAC1 and BAC2), the modified BGM clones (Tg-110CIBS and Tg-220CISP) and the fused BGM clone (Tg-250SB). The read mapping results are summarized in Table 1. No mutation was observed in the cloned and modified mouse genomic inserts during manipulations of the BGM vector, and all targeted modifications were accurately introduced. A few nucleotides differed from the reference sequence data in both BAC1 and BAC2. Two single-nucleotide differences were found between BAC1 and the reference sequence [GenBank: AC132096.4]: the replacement of G at positions 10349 and 113530 by A and T, respectively. Four differences were found between BAC2 and the reference data [GenBank: AC102535.17]: G at 210780, G at 210788, A at 210815 and G at 210840 were replaced by A, A, G and A, respectively. Therefore, starting from targeted insertion, all possible genetic modifications, i.e., insertion, deletion, inversion and fusion to the targeted site, could now be introduced using the BGM system without leaving unnecessary sequences in the insert DNA.

**Table 1:** Summary of the read mapping in DNA sequencing

	**BAC1**		**BAC2**		**Tg110CIBS**		**Tg220CISP**		**Tg250SB**	
	**# reads**	**%**	**# reads**	**%**	**# reads**	**%**	**# reads**	**%**	**# reads**	**%**
Total reference length	114,183		220,003		115,372		222,532		254,658	
Mapped reads	8,341,236	91.1	8,604,424	95.9	1,028,479	2.7	1,737,127	5.2	504,510	5.8
Unmapped reads	814,824	8.9	370,790	4.1	37,166,207	97.3	31,564,383	94.8	8,227,736	94.2
Reads in pairs	8,327,110	91.0	8,594,722	95.8	960,578	2.5	1,714,062	5.2	498,152	5.7
Broken paired reads	14,126	0.2	9,702	0.1	67,901	0.2	23,065	0.1	6,358	0.1
Total number of reads	9,156,060		8,975,214		38,194,686		33,301,510		8,732,246	
Total read length (Mb)	915.6		897.5		3,819.5		3,330.2		873.2	
Fraction of reference covered	1.00		1.00		1.00		1.00		1.00	
Minimum coverage	241		125		28		22		6	
Maximum coverage	15147		5631		2211		1514		574	
Average coverage	7100.7		3812.3		851.5		752.2		191.0	

Reads obtained from the Illumina Genome Analyzer IIx were analyzed and mapped to each reference sequence using CLC Genomics Workbench 5.5 (CLC Bio). The number of mapped reads in each sample was enough to evaluate mutations. Analyzed DNA samples are follows: original BAC clones of BAC1 and BAC2 and BGM clones of Tg110CIBS, Tg220CISP and Tg250SB.

### Generation of BGM transgenic mice

**Figure 6:**
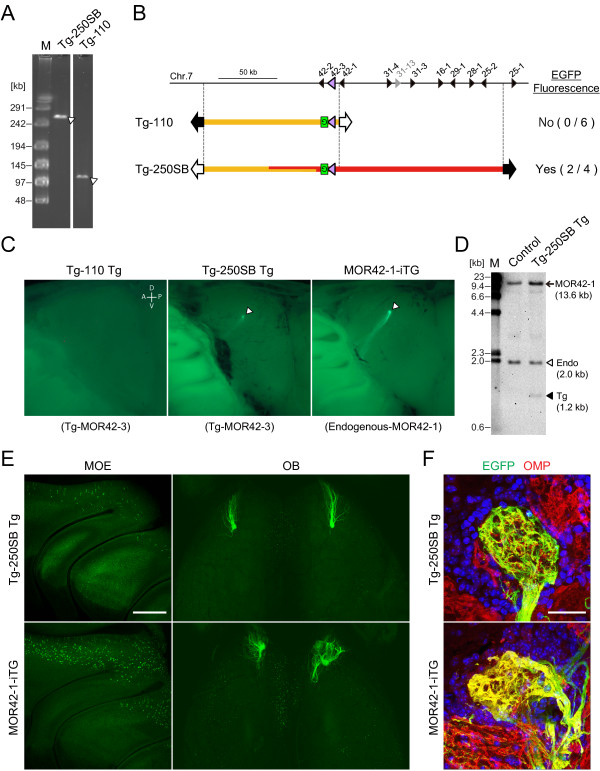
**Generation of transgenic mice and expression of the *EGFP *reporter gene.** (**A**) The quality and yield of the purified transgenes for microinjection were confirmed by CHEF. All signals indicate intact fragments and sufficient amounts for microinjection. The concentration of retrieved transgenes ranged from 0.3 to 3 ng/μl. (**B**) Schematic diagram of Tg-110 and Tg-250SB. Tg-250SB transgenic mice express the *EGFP* reporter gene, whereas Tg-110 transgenic mice do not. The number of EGFP-positive independent founders and lines among the total analyzed is shown in parentheses. (**C**) Whole-mount images of the medial aspect of the OB in transgenic Tg-110 (line #10, left panel) and Tg-250SB mice (line #8, medial panel) and the MOR42-1-iTG [28] gene-targeted mouse (right panel). Tg-110 transgenic mice do not show EGFP fluorescence, whereas Tg-250SB transgenic mice do. EGFP-labeled axons in Tg-250SB converge on the dorsal side of the OB and form a glomerulus. Arrowheads indicate glomeruli. D, dorsal; V, ventral; A, anterior; P, posterior. (**D**) Southern blot analysis of Tg-250SB line #8 and its transgene-negative littermate (control). The *MOR42-3* probe detected the endogenous (2.0 kb, open arrowhead) and the transgenic (1.2 kb, closed arrowhead) *MOR42-3* genes, and also detect both endogenous and transgenic *MOR42-1* gene (13.6 kb, arrow). (**E**) Confocal images of Tg-250SB line #8 transgenic and MOR42-1-iTG gene-targeted mice. Mosaic patterns of *EGFP*-expressing OSNs in the dorsal epithelium (medial view, left panel) and glomeruli formed by axons in the OB (dorsal view, right panel) were observed. (**F**) Tg-MOR42-3 (left panel) or endogenous-MOR42-1 glomeruli (right panel) were detected by immunostaining coronal cryosections. Red, OMP immunoreactivity. Green, EGFP immunoreactivity. Blue, DAPI nuclear staining, which marks the glomeruli structure. The glomeruli in the Tg-250SB transgenic mouse showed an intermingling of red (EGFP-negative) and yellow (EGFP-positive) axons, whereas the glomeruli in the homozygous MOR42-1-iTG mouse were fully doubly labeled. Scale bars: E, 500 μm; F, 30 μm.

Other cloning tools for large DNA fragments, such as BACs and YACs, in combination with transgenesis, have greatly contributed to life science research, including functional analyses of genomic DNA, the study of gene regulation mechanisms and disease research [3,25,26]. However, the BGM vector system has not yet been applied to transgenesis. Thus, we attempted to generate transgenic mice carrying the modified and reconstructed transgenes, Tg-110 and Tg-250SB, to demonstrate the potency of the BGM vector system in transgenesis (Figure 6). In contrast to other systems, the BGM vector system utilizes the entire endogenous genome as a vector. Therefore, the transgene can be obtained by digesting the genome with I-PpoI, whose recognition sites are introduced to flank the BAC vector sequence (Figure 1A). But its concentration is expected to be low because of the single copy of the transgene per genome/cell. Thus, we prepared linearized transgenes by excision from the BGM vector using I-PpoI digestion and CHEF and concentrated the transgenes by conventional electrophoresis followed by electroelution and dialysis with a high-salt injection buffer (Figure 6A). Using the method established in this study, linearized microinjection-grade transgenes were readily prepared from the BGM clones. The purified transgenes were microinjected into fertilized mouse eggs to obtain founder transgenic mice. New born mice were genotyped by PCR to amplify transgene-specific sequences. We designed three primer sets to amplify BAC vector sequences (left and right end of the transgene) and *EGFP* (middle of the transgene) to assess the integration of the transgene into chromosome. Assuming that all three PCR positive mouse would carry an intact transgene, we obtained six Tg-110 founders from 45 newborn mice. Partial integrations of the transgene were also observed in 2 mice, which were PCR positive for the left end of the transgene only. For Tg-250SB, four of 26 pups carried an intact transgene, and one lacked the left end sequence. These transgenic ratios are comparable to a conventional BAC transgenesis [3]. Germline transmission of the transgenes was obtained in 5 transgenic founders for Tg-110 (from 6 founders) and two for Tg-250SB (from 4 founders).

### Experimental evidence of a *cis-*acting element for a class I OR gene

An individual olfactory sensory neuron (OSN) expresses only one OR gene in a monoallelic and mutually exclusive manner [27]. Class I ORs are expressed almost exclusively by OSNs in the dorsal main olfactory epithelium (MOE), and these OSNs project their axons to a specific subset of glomeruli in the dorsal domain of the olfactory bulb (OB) [28,29]. The transgenes of the *MOR42-3* locus are designed to activate the bicistronic expression of *MOR42-3* and *tauEGFP*, a fusion of the microtubule-associated protein tau with EGFP, to visualize the *MOR42-3* transgene expression and OSN axonal projections. Thus, if the transgenes contain a *MOR42-3 cis*-acting element, *MOR42-3* expression can be monitored by EGFP fluorescence. For Tg-110, we analyzed 5 transgenic lines and 1 founder. None of the Tg-110 transgenic mice displayed labeled OSNs. For Tg-250SB, two transgenic lines established from two founders were EGFP positive (Figure 6B), indicating expression of *MOR42-3* transgene. Remaining two founders were EGFP negative. Whole-mount images of the MOE and the OB of Tg-250SB showed a punctate EGFP expression pattern within the dorsal region of the MOE and axonal projections into the dorsomedial and dorsolateral glomerulus of each OB (Figure 6C and E). To assess the copy number and integrity of the transgene, we analyzed Tg-250SB by Southern blot using *MOR42-3* coding region as a probe (Figure 6D), which cross-hybridizes to highly homologous gene *MOR42-1* (97% identical). Southern blot analysis showed that the specific band corresponding to the transgene was observed in expected size and the bands corresponding to endogenous *MOR42-3* were the same between control and Tg-250SB, indicating that the intact transgenes were integrated into chromosome, and this is not due to a gene targeting event. Ratio of signal intensities (transgene/endogenous gene) were 1.05 for line #5 (n = 1) and 0.46 ± 0.02 for line #8 (n = 4), indicating the copy number of transgene of two and one for line#5 and line#8, respectively. Because the Tg-250SB transgene carries *MOR42-1*, intensities of the band corresponding to *MOR42-1* increased 1.61 ± 0.18 fold in Tg-250SB line #8 with reference to the control, confirming one copy of the transgene was integrated.

OSNs expressing highly homologous ORs tend to project their axons to near but distinct subsets of glomeruli in the OB [30]. Because MOR42-3 and MOR42-1, members of the MOR42 subfamily, share 97% amino acid similarity in their coding sequences, the axonal projection site of *MOR42-3*-expressing OSNs was expected to be close to that of *MOR42-1*. Using axonal projection of OSNs expressing endogenous *MOR42-1* as control, we examined the MOR42-1-iTG gene-targeted mouse [28] in which an *IRES-tauEGFP* reporter was inserted downstream of *MOR42-1*. The glomerular positions of transgenic-MOR42-3 (Tg-MOR42-3) in Tg-250SB transgenic mice were similar to those of endogenous MOR42-1 (Figure 6C, E). We further analyzed the detailed structure of the Tg-MOR42-3 glomeruli. Glomeruli are innervated exclusively by axons from OSNs expressing the same OR [31]. We performed double-label immunohistochemistry on coronal cryosections with antibodies against olfactory marker protein (OMP) to stain all mature OSN and antibodies against EGFP to label *MOR42-3* transgene- or *MOR42-1* endogenous gene-expressing OSNs. Tg-MOR42-3 glomeruli contained a mixture of EGFP-negative and EGFP-positive axons, whereas MOR42-1 glomeruli of homozygous MOR42-1-iTG mice were completely EGFP positive (Figure 6F), suggesting that axons of OSNs expressing Tg-*MOR42-3* formed glomeruli together with those of OSNs expressing endogenous *MOR42-3*[32]. The projection site and converged glomerular structure of OSNs expressing the EGFP reporter suggest proper expression of the *MOR42-3* transgene. Taken together, these results indicate that a *cis*-acting element for *MOR42-3* is present in the extended region from Tg-110 and provide the first experimental evidence of a *cis-*acting element for a class I OR gene.

## Discussion

The BGM vector system is a unique and developing technology for propagation of very large fragments of heterologous DNA. In this study, we have demonstrated that the BGM vector system enables the complete genetic modification of large genomic DNA fragments, including targeted insertion, deletion, inversion and fusion. In addition, we demonstrated the existence of a *cis*-acting element of a mouse class I OR gene by combining the BGM vector system with mouse transgenesis.

The BGM vector has several specific features that create advantages over the BAC and YAC systems. First, the megabase-scale cloning capacity of the BGM vector is greater than that of conventional systems. The BGM vector system is capable of cloning the entire 3.5 Mb *Synechocystis* genome [15], and the upper limit of clone size has not yet been determined. Second, cloned DNA inserts show high structural and genetic stability [10] because of their direct insertion into the single circular host genome. In fact, DNA sequencing of modified and reconstructed genomic DNA fragments confirmed the structural stability of inserts in the BGM vector even though genome inserts included many repetitive sequences and several similar class I OR genes. However, it should be noted that, similar to the YAC system, recombination is active in *B. subtilis*; thus, unwanted rearrangements may occur. This issue can be solved by introducing an inducible RecA system into the BGM vector. Third, various accurate modification approaches are available. The *c*I repressor cassette-mediating modification technique provides desired gene modifications without leaving any traces in the DNA, enabling the repetitive modification of BGM inserts. In addition to insertion and deletion modifications, we demonstrated the elongation of inserts via the fusion of two DNA fragments even though they were initially oriented in opposite directions in the BAC vector, thus providing a method for the construction of giant recombinant DNA fragments. Considering with maximum cloning capacity of the BGM vector of 3 Mbp [15], the fusion of contiguous genomic fragments is a powerful technique for the reconstruction of gene structures surrounding intergenic regulatory elements [16,33]. In addition, our sequencing analyses confirmed that these targeted genetic modifications were accurate and reliable. Fourth, BGM inserts can be simply retrieved by I-PpoI digestion because a single host cell contains a single genome composed of the recombinant insert and the 4.2 Mb BGM vector. As we demonstrated, BAC clones are easily transferred, modified and reconstructed in the BGM vector. It is noteworthy that modified BGM inserts can be restored to a circular BAC form [10], enabling this “shuttle genetic modification” of BAC clones to enhance the utility of BGM vector system.

Finally, we have used mouse genomic DNA to demonstrate the suitability of the BGM system for genetic manipulation and transgenesis. Many BAC libraries have been already established and/or are under construction for diverse species, including mammals, other vertebrates and plants [34]. Because the BGM vector harbors BAC vector sequences for cloning BAC inserts, this system can be applied to other species. Moreover, the BGM vector can be designed to clone other library resources by introducing cloning vector sequences from other systems, e.g., YACs and human artificial chromosomes. Because the BGM vector system can provide large cloning capacity in size and various accurate gene manipulation approaches, the BGM vector system is an attractive cloning tool for the manipulation of large DNA fragments, such as in the functional analysis of genomic DNA and recombinant genomes in synthetic biology [9,35].

## Conclusion

We demonstrated targeted insertion and inversion methods in the BGM system to add to its repertoire of genetic modification approaches. Using these techniques, a 252 kb transgene was reconstructed from two BAC clones whose inserts were initially oriented in opposite direction with reference to the BAC vector sequence. DNA sequence analysis of modified BGM clones demonstrated the genetic stability of inserts and correct modifications. Furthermore, we established and applied BGM-based mouse transgenesis. By analyzing the generated transgenic mice, a *cis*-acting element for a mouse class I OR gene was experimentally demonstrated for the first time. The BGM vector is a new platform that provides a complete genetic modification approach for large genomic DNA fragments without leaving selection markers or dispensable sequences. The BGM vector system and its application to transgenesis offer a new genetic approach for not only systems and synthetic biology but also other life science research fields.

## Methods

### Strains and preparation of competent *B. subtilis*

The *B. subtilis* strains and the BAC-specific BGM vectors BEST310 and BEST6528 [10,16] were kindly provided by Dr. Itaya. These strains are derived from the restriction modification-deficient strain RM125 to possess a *c*I repressor gene cassette flanked by pBAC108-based BAC vector sequences [1,10] and are identical with the exception that BEST6528 contains a 100 kb spacer sequence and an additional *c*I repressor gene cassette [16]. For defining insert orientation, the half of the BAC vector containing the chloramphenicol resistance gene for *E.coli* is defined as right side (open arrows in Figures). The preparation of competent cells and transformation of *B. subtilis* were performed as described elsewhere [12]. Strains containing multiple antibiotic-resistance genes were tested using the replica plating method. *B. subtilis* was routinely grown in 1–5 ml Luria-Bertani (LB) broth at 37°C by rotating (>50 rpm) or shaking (200 rpm). Antibiotic Medium 3 (Difco) was used for the selection of the BGM transformants with the following antibiotics, as appropriate: neomycin (Nm, 5 μg/ml, Sigma), spectinomycin (Spc, 50 μg/ml, Sigma), erythromycin (Em, 5 μg/ml, Sigma), phleomycin (Phl, 0.5 μg/ml, Sigma), tetracycline (Tet, 10 μg/ml, Nacalai) and blasticidin S (Bs, 250 μg/ml, Funakoshi).

### One-step transfer of BAC inserts to the BGM vector

Two contiguous BAC clones, RP24-392H7 and RP23-61O11 (designated BAC1 and BAC2, respectively), were purchased from the Children’s Hospital Oakland Research Institute. The BAC DNA was prepared by the alkaline lysis method and subsequent equilibrium centrifugation in a CsCl-ethidium bromide gradient [17]. BGM strains were transformed with the purified BAC DNAs [10]. BAC1 and BAC2 were cloned into BEST310 and BEST6528, respectively.

### I-PpoI/CHEF analysis

The cloned inserts in the BGM vectors were analyzed by I-PpoI digestion followed by CHEF electrophoresis. Agarose plugs containing BGM clones were prepared as described [12]. A sliced block of the agarose plug was soaked in 200 μl of I-PpoI digestion buffer for 15 min to replace the TE buffer in the agarose plug. After discarding the soaking buffer, the block was immersed in 100 μl of digestion buffer containing 20 U of I-PpoI (Promega) and incubated for 1–1.5 hours at 37°C. The plug containing the digested DNA was embedded in a well of a 1% (w/v) agarose gel and separated by CHEF (Bio Craft) in 0.5 × TBE buffer (50 mM Tris–borate (pH 8.0), 1.0 mM EDTA). Electrophoresis was performed at 4 V/cm at 14°C under the following conditions: 12 sec for 21 h (BAC1, Tg-110), 30 sec for 22 h (BAC2, Tg-220) and 25 sec for 22 h (Tg-250SB).

### Southern blot analysis of the BGM clones

Genomic DNA from the BGM clones was prepared using the liquid isolation method [12]. The genomic DNA was digested with EcoRI, HindIII or BamHI (TaKaRa). The digested DNA was separated in pulse-field gels at 3 V/cm, 18 sec for 14 h at 14°C, and the DNA was transferred onto a Hybond-N (GE Healthcare) membrane filter. The preparation of digoxigenin (DIG)-labeled DNA probes, Southern hybridization and detection with NBT/BCIP were performed using a DNA labeling and detection kit (Roche).

### Construction of plasmids

To construct the reporter cassette insertion plasmid, a linker containing AscI, SpeI, BglII, NdeI and SphI sites was inserted between the XbaI and SacI sites of pBluescript II SK(+) (Stratagene) to construct the pT1 vector. An EcoRI-XbaI fragment of *IRES-tauEGFP* from the *iTGFP-ACNF* plasmid [23] was cloned into pT1 to generate pT1-iTGFP. The 1.0 kb left (L) and right 1.0 kb (R) arms for the targeted insertion of *IRES-tauEGFP* into the 3 bp downstream of the *MOR42-3* stop codon were prepared by PCR and contained sequences that were homologous to the upstream and downstream *MOR42-3* insertion sites, respectively. The L arm was first cloned into the SalI-EcoRI site of pT1-iTGFP, and then the R arm was cloned into the SpeI-BglII site to generate the plasmid piTG423. The piTG423-CISP plasmid was constructed by inserting a selection marker-containing the *c*I-*spc* cassette into the AscI-SpeI site of the piTG423 plasmid.

To construct the inversion plasmid, the new BAC plasmid p108IPpoI-HPNSB was generated by inserting a linker containing PmlI, NotI and SphI sites between the HindIII and BamHI sites of the BAC plasmid p108NHBN-MIM [10]. The *te-erm* cassette, which was obtained from a NotI-digested fragment from pBEAZ191 [24], was inserted into the PmlI site of this plasmid after blunt-end treatment to generate the plasmid p108Term. Similarly, the *phl-et* cassette, a NotI fragment from pBEAZ195 [24], was inserted to generate the plasmid p108Phlet. A 0.9 kb fragment homologous to the left end of Tg-110 was cloned into the NotI site of p108Term. A 0.8 kb fragment homologous to the right side of Tg-110 (1.3 kb from the right end) was then cloned into the HindIII site of p108Phlet.

Fusion plasmids were also constructed based on p108IPpoI-HPNSB. The p108CISP plasmid was constructed by inserting the *c*I-*spc* cassette, a HindIII-BamHI fragment excised from pCISP310B, into the PmlI site after blunt-ending. Similarly, p108CIBS was constructed by cloning the *c*I-*bsr* cassette, a PstI fragment derived from pBEST10007. A 1.3 kb PCR fragment homologous to the left end of Tg-220 was cloned into the NotI site of p108CISP, and a 1.3 kb fragment homologous to the right end of the inverted Tg-110 was cloned into the MluI site of p108CIBS.

All of the plasmids were linearized with the appropriate restriction enzymes (TaKaRa or Toyobo) and used for transformation. The all homologous arms (approximately 1 kb) and *c*I-*spc* cassette were amplified by PCR (PrimeSTAR HS DNA polymerase, TaKaRa, or KOD plus DNA polymerase, Toyobo) using the BAC clones and pCISP310B [10] as templates, respectively. Primer sequences and PCR conditions are summarized in Additional file 2. The orientations of the inserts were determined by restriction enzyme digestion or PCR. The accuracy of the sequences generated by PCR was confirmed by DNA sequencing.

### DNA sequencing

Sequences of the original BAC and modified BGM clones (Tg-110CIBS, Tg-220CISP and Tg-250SB) were verified using next-generation sequencing. Briefly, genomic DNA (3 μg) from the above clones was fragmented to an average length of 300 bp using the Covaris S2 system (Covaris). After purification, end-repairing, A-tailing, paired-end adapter ligation and 12-cycle PCR were performed using the NEBNext DNA Library Prep Master Mix Set and NEBNext Multiplex Oligos for Illumina (New England Biolabs). All libraries were quantified using an Agilent Bioanalyzer 2100 (Agilent Technologies) and pooled to provide equal genome coverage from each library. Pooled libraries were sequenced in a single lane of the Illumina Genome Analyzer IIx (Illumina), which produced 102 paired-end reads, in accordance with the manufacturer’s instructions.

Reads obtained from the Illumina Genome Analyzer IIx were analyzed using CLC Genomics Workbench 5.5 (CLC Bio). Reads were trimmed and mapped to each reference sequence with default parameters. The reference sequences used in this study included the following: BAC1, original BAC clone RP24-392H7 [GenBank: AC132096]; BAC2, original BAC clone RP23-61O11 [GenBank: AC102535]; B1TgCIBS and B2TgCISP, predicted modified mouse genomic Tg-110CIBS and Tg-220CISP insert sequences; and Tg250SB, a predicted fused mouse genomic Tg-250SB insert sequence. The mapping results are detailed in Table 1. After mapping the reads, variant calling was performed using the probabilistic variant detection function with default parameters, and variants with frequencies of at least 90% were considered. All read data have been deposited in the DDBJ Sequence Read Archive (DRA) under accession number [DRA000859].

### Preparation of the transgenes for pronuclear injection

Genomic DNA carrying the transgene in an agarose plug was prepared, digested with I-PpoI and resolved by CHEF in a 1% (w/v) agarose gel using sterile 0.5 × TBE, as performed for the I-PpoI analysis. To concentrate the transgene fragment, the band was excised from the agarose gel, turned vertically and embedded in 4% (w/v) agarose; electrophoresis was then performed at 3.3 V/cm for more than 10 hours in 0.5 × TBE. The excised concentrated transgene band was placed in a prepared dialysis tube hydrated with 0.5 × TBE, and the transgene was electroeluted from the gel under the same conditions. The eluate was dialyzed with injection buffer (10 mM Tris–HCl (pH 8.0), 0.1 mM EDTA, 100 mM NaCl) at 4-6°C overnight. The concentration and integrity were estimated based on the control band intensity in the CHEF analysis. The purified transgenes were used immediately. Alternatively, 75 μM spermidine and 30 μM spermine were added for long-term storage. The stock transgenes were diluted to 0.3-1.5 ng/μl with injection buffer before use.

### Production and analyses of transgenic mice

The purified transgenes were microinjected into the pronucleus of B6C3F1 (C3H/HeSlc male × C57BL/6NCrSlc female) mouse zygotes. Injected eggs were transferred to the oviducts of pseudopregnant female ICRs. The founders were screened by PCR with the following three primer sets that specifically amplified the internal and left and right ends of the transgenes: EGFP: GFP-F, 5′-GGCATCAAGGTGAACTTCAAGATCC-3′ and GFP-R, 5′-CTTTACTTGTACAGCTCGTCCATGC-3′; the left end of the BAC sequence: SacB-F, 5-GCTGAATACAACGGCTATCACG-3′ and SacB-R, 5′-TCTCTCAGCGTATGGTTGTCG-3′, or BAC108 L-F, 5′-CGTATTCAGTGTCGCTGATTTG-3′ and BAC108 L-R, 5′-TTAGCGATGAGCTCGGACTTC-3′; and for the right end of the BAC sequence: CmR-F, 5′-GAGGCATTTCAGTCAGTTGCTC-3′ and CmR-R, 5′-CGGCATGATGAACCTGAATCG-3′. All founder mice positive for these three primer sets were selected as candidates containing intact transgenes. The founder mice were crossed with C57BL/6 mice. The transgenic mice were dissected and fixed in 4% paraformaldehyde (PFA)/PBS for 10–15 min on ice. Line 8 of two EGFP-expressing Tg-250SB transgenic lines was used for the expression analyses in Figure 6.

For Southern blot analysis, 10 μg of genomic DNA extracted from a tail was digested with EcoRI (TaKaRa) and separated on a 1% agarose gel. The DNA was transferred onto a membrane filter, and hybridized with a DIG-labeled *MOR42-3* coding probe (nucleotides 190 – 1091 from NM_020289). The signals were detected by chemiluminescence (CSPD, Roche) using a CCD camera (ChemiDoc™ XRS, Bio-Rad). The copy numbers were estimated by comparing the intensities of transgenic and endogenous signals.

For immunohistochemistry (IHC), mice were fixed by perfusion with 4% PFA/PBS and infiltration in the same fixative solution at 4°C for 30 min. After cryoprotection with 15% and 30% sucrose/PBS, tissues were embedded in Frozen Section Compound (Surgipath FSC22, Leica microsystems). Serial cryosections (20 μm) were collected on MAS-coated microscope glass slides (Matsunami) and dried for 1 h at room temperature. IHC was performed by a standard protocol [36]. After post-fixation, permeabilization and antigen-retrieval pretreatment, sections were blocked with 10% normal horse serum and incubated with primary antibody at 4°C overnight. The following primary antibodies and dilutions were used: rat anti-GFP (1:2000, catalog #04404-84, Nacalai) and goat anti-OMP (1:5000, catalog #544-10001, Wako). After incubation of the primary antibodies, sections were washed in PBS containing 0.01% Tween 20 and stained by the following Alexa Fluor-conjugated secondary antibodies (1:500, Invitrogen): Alexa Fluor 488-conjugated donkey anti-rat IgG and Alexa Fluor 546-conjugated donkey anti-goat IgG. Nuclear staining was performed with DAPI (1:1000, Vector Laboratories), and CC/Mount (Diagnostic BioSystems) was used for mounting.

Fluorescent images of endogenous GFP and IHC signals were taken with Olympus SZX10 fluorescent stereomicroscope with a DP71 digital CCD camera and Leica SPE confocal microscope. Confocal images were collected as *z*-stacks and projected into a single image for display. Images were analyzed and adjusted using Photoshop CS4 (Adobe). All of the mouse studies were approved by the Institutional Animal Experiment Committee of the Tokyo Institute of Technology and performed in accordance with institutional and governmental guidelines.

## Abbreviations

BAC: Bacterial artificial chromosome; BGM: *Bacillus subtilis* genome; CHEF: Contour-clamped homologous electric field; EGFP: Enhanced green fluorescent protein; MOE: Main olfactory epithelium; OB: Olfactory bulb; OR: Odorant receptor; OSN: Olfactory sensory neuron; YAC: Yeast artificial chromosome.

## Competing interests

The authors declare that they have no competing interest.

## Authors’ contributions

TI, SK and JH designed and performed the research. YS and HY performed the DNA sequencing. TE performed immunohistochemistry. TI and JH wrote the manuscript. All authors read and approved the final manuscript.

## Supplementary Material

Additional file 1 **Efficiency of cloning BAC inserts into the BGM vector.** This file contains the efficiency of cloning BAC clones into the BGM vector: clone names and ID, length of BAC inserts, BGM vector used for cloning and the numbers of tested and insert-positive clones. Click here for file

Additional file 2 **Primer sequences and PCR conditions.** This file contains information of all primer sequences and PCR conditions used in this study for constructing homology arms, cassettes and new BAC plasmid, screening of BGM clones and genotyping. Click here for file
